# Mania Ephemera

**Published:** 1863-01

**Authors:** J. Crichton Browne

**Affiliations:** Assistant-Physician Derby County Asylum.


### Art. IV.—MANIA EPHEMERA.

By J. Crichton Browne, M.D., Assistant-Physician Derby County Asylum.

Notwithstanding the vaunted intelligence of the age in which we live, the education of the masses, the mental illumination of the upper ten thousand, the universal dissemination of correct views and sound principles, a pestilential atmosphere of ignorance and error is still somehow and somewhere engendered, is still freely circulating through the magnificent and glittering social edifice and condensing itself especially in some mysterious corners. In connexion with insanity and the insane, errors both of fact and of sentiment are even yet peculiarly prevalent. While relatives can stand round, and with smiling complacency contemplate the arthritic throes of some antiquated *bon vivant,* whose every joint is in a state of chalky degeneration, who is but a mass of tophitic monuments, piled up to the memory of "departed joys" of the table and unrestrained appetites; they will turn away with looks of horror and aversion from regarding the wanderings of some noble, but disordered mind, overthrown from its very loftiness and from the elegance of its structure, which rendered it unable to withstand the rude blasts of a tempestuous world. A man will proclaim that he has been a martyr to gout, but he will never confess that he has been the victim of mania. A *genteel* family is not more under the ban of disgrace when one of its members is enjoying the *otium cum dignitate* of a convict establishment, than when one is in an asylum. This, indeed, is the culminating point and highest dishonour; for what can more effectually degrade a man and ruin his prospects than residence in an institution set apart for the treatment of insanity? In the public eye it is bad enough to have been insane at home, but it is tenfold worse to have sought seclusion and those means most approved as contributing to cure. He who has once crossed the threshold of an asylum is popularly considered good for nothing afterwards; and even many of those who pride themselves on their enlightenment, and who would theoretically repudiate such a doctrine, practically give in their adherence to it, by refusing to take into their service or to place confidence in the discharged lunatic. I have myself known an instance, in which a friendless girl, recovered from an attack of mania, has gone forth from an asylum, has fruitlessly sought employment, has found no rest for the sole of her foot, and has at length been driven to hide her misfortune in "the dark flowing river."

A discharge from an asylum is thus to some lunatics, a positive calamity, implying as it does, the loss of a comfortable home and kind friends, and a return to toil and anxiety, to a world that has affixed a stigma to the affection from which they have suffered, and that regards with suspicion and distrust any one who has come out of a "mad-house."

It is assuredly the duty of the medical profession to wage unceasing and determined war against those popular errors and prejudices regarding insanity and asylums, which we have just indicated; which are so unfounded, so injurious, and so calculated diminish the chances of cure, by inducing patients and their relations to conceal insanity in its incipient stages, when it is most susceptible of benefit by treatment, and to procrastinate removal to an asylum, until such removal is useless—until, indeed, what might have been only a transient defect has become a lifelong deformity. But it is also the duty of medical practitioners to allow themselves to be regulated to a certain extent by these prejudices, and to protect their patients, as far as may be, from the evil effects of popular prepossessions, which will not be uprooted and consumed in a day nor a year. It therefore becomes a matter of the highest importance to distinguish at once those cases in which incarceration in an asylum is demanded and to which a certain amount of publicity must therefore be given, from those others which may be treated with equal success in their own homes and the nature of which may be altogether concealed. A very serious responsibility devolves upon each medical man who is called upon to pronounce judgment in a case of mental disease. If, without sufficient cause, he detains his patient amongst his family and friends, surrounded by the very circumstances which may have awakened his morbid ideas, and deprives him of the restorative influences of new and unexpected impressions and of all those appliances and measures which are only to be found in a well ordered asylum, he must be harassed by the reflection that he may have been instrumental in impairing or retarding recovery. And if on the other hand, with ill-advised precipitancy, he has hurried off his patient to seclusion, during some transitory aberration of mind, he will afterwards doubtless have the distressing consciousness that he has injured the social position and prospects of one whom he only desired to benefit. Of the two errors in practice referred to, the former is certainly the more common, but the latter is also of occasional occurrence; and several cases which have been lately brought under my observation have directed my attention to a form of mental disease in which such a mistake is not unlikely to be committed. I allude to ephemeral mania, which consists in a transitory isolated attack of mental disturbance, usually not exceeding forty-eight hours in duration, and which is apt to be confounded with ordinary general mania—which malady it very closely resembles in many particulars. The brevity of its continuance, however, separates it widely from mania proper, and renders it quite unnecessary that those suffering from it should be removed to an asylum. Indeed, any such removal would be prejudicial to those afflicted with mania ephemera; for upon recovering themselves and awakening as from a troubled dream, they would obviously incur great risk of relapse or of regression into some more permanent affection from the shock, at finding themselves in such a place, from the vexation and chagrin inseparable from a realization of their true position. It is therefore, of momentous consequence to recognise this disorder, which is capable of easy cure at home, which is so fleeting and evanescent, and in which the mind is not overthrown nor even gravely damaged; for whenever the tyranny of the attack is overpast, with wondrous elasticity the mind springs up and regains its former stature and rectitude like "a wind-bent flower released." No differential diagnosis, however, between ephemeral and ordinary mania, as yet exists. This can only be attained in a serviceable and accurate form by a careful observation and record of cases, and by inferences drawn from these; and it is as a trifling contribution to this very desirable object that the present paper has been written.

Every species of insanity may manifest itself in the form of a short temporary attack, but such attacks are of rare occurrence in every variety except mania. In this statement isolated hallucinations, recognised as such, and experienced by persons of sound mind, are not of course included, as they can scarcely be regarded as instances of insanity in the common acceptation of that term. But leaving these out of consideration, there can be no doubt that acute mania is the kind of insanity most often met with in the form of a short solitary paroxysm. In what is said, however, regarding this affection in this paper, all notice of one class of ephemeral maniacal paroxysms is purposely omitted. No allusion is made to mania epileptica, to those temporary attacks of excitement which often immediately precede or follow epileptic seizures. The frequent recurrence of these attacks, their violent, sometimes desperately homicidal character, imperatively demand that care and treatment which an asylum alone furnishes, and place them beyond the scope of the present inquiry.

The causes of mania ephemera are very various. Debilitating influences, such as deficient nourishment, impurity of the atmosphere, sedentary habits, confinement, and lack of exercise, exhausting exertion of mind or body, but especially of the former, unhappy circumstances, previous disease of a weakening or nervous character, excessive indulgence in stimulants, and hereditary proclivity to disease, are all powerful in predisposing to it, as to disease in general. These causes are particularly potent when operating upon a nervous temperament. Excessive mental emotion is pre-eminently an exciting cause. Grief, surprise, fear, anger, or joy, is a usual precursor. A man may be frightened to madness as well as to death. This madness may be of no temporary kind, but it is nevertheless true that the great majority of instances of temporary insanity may be traced to overwrought feeling in its corporeal relations. It is indeed characteristic of ephemeral mania that its immediate cause is almost invariably obvious and prominent, and not, as frequently is the case in other varieties of mental alienation, hidden and imperceptible, quietly, stealthily undermining bodily functions and mental powers, unrecognised even by watchful onlookers, except in the catastrophe which it has brought about. It is further characteristic that the cause is generally quick and sudden in its operation, not slowly progressive, but rapidly culminating in mental derangement. The more violent passions, ungoverned bursts of temper, unexpected sorrows, family dissensions, reverses of fortune, bitter disappointments, agitating joys, novel and powerful religious impressions, are most prolific exciting causes, and these acting in combination with certain predisposing and physical circumstances, bring about a temporary perturbation of mind, just as with predisposing and physical circumstances of another description, they might entail more lasting disorder. Indeed, ephemeral mania seems generally to consist essentially in an alteration of the cerebral circulation, following upon some kind of over-excitement of the emotions. It is sometimes a blushing of the brain after a profound emotion.

The application of an appropriate stimulus to any organ causes a determination of blood to it. Snuff ensures a flow of blood to the nose, spices to the salivary glands, food to the stomach, diuretics to the kidneys. The amount of vital turgescence in such instances is determined by the degree and continuance of the stimulation. The well-being of a part, or its function, being menaced by any unusual excitement, more blood is required to supply unusual loss and to perform other conservative services. The arteries thus become dilated and transmit their contents with augmented velocity, the circulation is increased, and the result is, that the nutrition and sensibility of the part are also increased if the stimulation be moderate, and are perverted if it be excessive in degree.

Now it is well-known that determination of blood to the brain does take place in certain persons, in consequence of mental excitement, as is shown by the violent throbbing of the carotid and temporal arteries, flushing of the face, giddiness, &c., which often follow cerebral stimulation. Even in healthy persons, or in persons of plethoric habit, this determination of blood may occasion transient delirium, with various signs of encephalic disturbance, such as extreme sensibility to light and sound, restlessness, pain in the head, and visual hallucinations. A flood of distorted ideas flows through the mind and overwhelms it; bewilderment and incoherence follow, and for the time being the patient is to all intents and purposes maniacal. A distinguished physician narrates the case of a gentleman subject to attacks of determination of blood to the head, "which caused him so much suffering and loss of moral control that he cut his throat to destroy his life. Whilst recovering from the wound, attacks sometimes came on, first with beating of the carotids, then with flushing of the face and head, suffusion of the eyes, and feelings of distraction in the head." But it is not in the plethoric or healthy that determination of blood to the brain is so likely to produce ephemeral mania as in the weak and anæmic, who, though suffering from general depression and debility, are still liable to irritation and exaltation of all the corporeal functions. And of all functions, those of the nervous centres have been found most liable to excitement in cases of spanæmia. The generally intensely nervous character of persons with greatly prostrated strength has been long remarked, as also their proneness to excitement. Dr. Williams has offered an explanation of this apparent anomaly. He states that the blood-vessels of the nervous centres, in consequence of their non-exposure to atmospheric pressure and their attachments to bony canals, do not, in spanæmia, shrink as the blood within them becomes reduced in quantity and quality. The disproportionate amount of blood which they thus contain, acts in accordance as it is affected by the heart's propulsive power, and thus, under the influence of emotion, excitement, or palpitation, the cerebral vessels receive an unusual share of increased but partial force as a result of their patency to the heart's action. An erethism of the functions of the nervous centres is the consequence of this, and thus mania ephemera may be produced by congestion in anæmia. When this is the case, with great heat and throbbing of the head, the trunk and extremities may remain cold and comparatively bloodless, just as with palpitation of the heart the pulse at the wrist may be almost imperceptible. But apart from congestion, or any discoverable circulatory change, mania ephemera may arise out of a mere preponderance of nervous action, in those remarkable for delicate nervous systems and strong innervation. This hyper-cœnæsthesis is most frequently exhibited in females at the period of development or at the climacteric period of life, and most in those who have been exposed to influences that heighten sensibility, weaken spontaneity, and tend to create a preponderance of the sexual feelings and relations.

The symptoms of mania ephemera vary greatly, and are commonly in intimate relation with the exciting causes of the attack. The patient generally appears as if he were partially drunk, or as if he were living and acting some confused and whimsical dream. He talks nonsense, utters meaningless ejaculations, breaks off in the midst of his sentences, makes ineffectual efforts to express his thoughts, wanders in a labyrinth of untold loveliness or of hideous grotesqueness, is tossed in a whirlpool of contending emotions, now buoyed up by hope and triumph, and again drawn downwards by terror and despair. He laughs and cries alternately, or he is irritable, capricious, or self-willed. He is impetuous in manner or violent, sometimes suspiciously avoiding those around him, sometimes attacking them with savage ferocity. He may be subject to hallucinations of all the senses and labour under an abnormal motor exaltation. Indeed, there is always a tendency to motor exaltation and rapid movement when the mind is agitated with strong emotion. The deranged mind delivers itself up, unrestrainedly, to this impulse, and the maniac rushes to and fro with a frantic recklessness, in harmony with his thoughts, climbs, attitudinizes, tears and destroys the articles around him.

In all this description, however, there is nothing which is not applicable to ordinary acute mania as well as to mania ephemera, and, indeed, it is no easy matter to discover distinctions between these two varieties which shall be universally or even widely correct. The individual cases often approach so closely to each other, that it would be impossible to point out diagnostic signs, while others again present points of difference which, if carefully observed and recorded, may ultimately guide to a more clearly defined line of demarcation.

In a large majority of cases of mania ephemera, there are none of those premonitory symptoms which so generally herald the approach of ordinary mania. The sufferer may be weak, anæmic, and in bad bodily health ; but until the occurrence of the exciting cause the mind most commonly remains unclouded. There is none of that impairment of judgment, that tottering of reason before her final fall, that feverish uneasiness, that morbid activity, that rash speculation, that inexplicable dread of impending calamity, that unwonted perverseness, or that terror-haunted sleeplessness, which so clearly betokens serious vascular disorder in the brain, and by which acute mania is so often preceded. So also, during the paroxysm, the involvement of the mind seems usually to be less deep and entire than in ordinary mania. No doubt in many cases, even of ephemeral mania, the mind is totally engulfed in the disorder ; but in others a certain degree of intelligence continues to preside over the wreck, and to impart some trifling guidance to it. The patient often appears to appreciate his position, to know that he is not himself, while he also recognises his friends, and distinguishes phantasms from realities. To certain types of the affection, as, for example, the ecstatic, this will not apply, as the mind is then altogether absorbed in the current of morbid ideas ; but it is at the same time true with reference to many instances, springing out of family brawls and disagreements, and other emotions not religious in their nature. In ephemeral mania there is generally more regard for cleanliness and decency than in mania proper. Those internal sensations of intolerable heat which prompt maniacs to denude themselves, and go about naked; those hallucinations of touch which induce them to expose their persons and bedaub themselves with filth and ordure, are absent; so modesty and propriety are not ordinarily violated. But this, also, is only a general rule, liable to many exceptions. The celebrated Dr. Fothergill, in an attack of mania ephemera, was seized with an uncontrollable desire to perambulate the streets of Edinburgh naked, preaching repentance. Another general observation is, that the language in ephemeral mania is not so blasphemous and obscene as in mania proper. When it is of a highly erotic character it will most frequently be found that the disease has originated in religious excitement. The destructive tendencies are commonly well marked in mania ephemera, and homicidal impulses are not unfrequently combined with it, thus rendering it a dangerous malady, and giving rise to many problems of the highest interest to the medical jurist, and eminently worthy of his most attentive consideration. In this disease, of all the animal instincts and passions destructiveness seems to hold the supremacy, and divested as the mind is of the capacity to act in compliance with the dictates of the higher sentiments, unbridled licence is given to any predominating impulse. Thus diabolical crimes are occasionally committed or attempted during an attack of ephemeral mania. The following example is from Marc. "Obs. 204.—A shoemaker, æt. 35, industrious and sober, rose early, and engaged in work. Very speedily his wife was struck by his incoherent observations and distracted expressions. The unfortunate man seized a knife and rushed upon her in order to kill her. The neighbours restrained the madman in order to prevent the catastrophe. He defended himself with the knife. His face was red; his pulse full and frequent; his tongue dry, and the surface of the body covered with perspiration. About noon he became calm and slept. In the evening he was perfectly natural and rational, but recollected nothing of what had passed."* Feuchtersleben relates the case of a young man, "in perfect health, who awoke suddenly one night in a fit of raving madness, ill-treated his wife, attempted to leap out of the window, and struck at whatever came in his way. An emetic put an end to this scene in an hour, since which he had been in a perfect state of health, never having had a recurrence of the attack." Many other examples might be adduced to illustrate the occasional supremacy of destructive instincts in mania ephemera; but I shall content myself with citing but one

* *De la Folie, considérée dans ses Rapports avec les Questions Medico-Judiciaires.* Par C. C. H. Marc. Tome ii. p. 510.

more, which came under my own observation, and which, moreover, illustrates several other points of interest in this disease.

F. F., æt. 50, a small farmer, of nervous temperament, was brought to the Derby County Asylum* in a strait-waistcoat, his legs being secured by ropes. His body was marked with several extensive bruises in consequence of the coercion to which he had been subjected. He was shouting aloud short incoherent sentences, uttering imprecations against those around, whom he seemed to suspect of conspiracy against him, and struggling violently to be free. He did not answer the questions put to him, but continued to cry out and to cast furtive glances about him. He appeared to be in weak bodily health, his pulse was a hundred, but feeble and thready, his face flushed, his head hot, his tongue coated with a white fur; respiration hurried. The pupils were slightly dilated, but sensitive to light, the muscular movements were tremulous. General and special sensibility were normal, as far as could be ascertained. The history of the case was elicited as follows. F. F. had suffered much grief and anxiety on account of the undutiful and cruel conduct of his children, also from business reverses; and after a domestic quarrel, twenty-four hours prior to his admission, suddenly became insane. His insanity was manifested by sudden maniacal excitement and incoherence. He had stripped himself to his shirt, seized a crowbar, rushed from his house and down the public street, threatening to murder any one who approached him. It was with the utmost difficulty he was overpowered and restrained. He had been sleepless and had continued raving wildly all night. He never was insane before, and never had any relatives insane. Immediately on his admission he had a warm bath and a mild purgative. He had not been an hour in the institution before he became comparatively rational. At his own request he retired to bed. He at once fell into a placid sleep, and on his awaking in the evening, no trace of insanity was discoverable in him. He has since continued quite well. He has but a very dim recollection of all that transpired during his excitement. The recovery of this man may have been merely contemporaneous with his removal to an asylum; but the novelty of his position, the influence of discipline, the helplessness and ineffectiveness of a single will, the absorption of personality in the general movement of a large establishment, and the order and quietness which prevail, may have been also instrumental in guiding his thoughts into their natural channel, and in recalling consciousness and self-control.

---

* I am indebted to Dr. Hitchman's kindness for permission to make use of cases admitted into this asylum.

The condition of general sensibility in mania ephemera is very similar to that in ordinary mania. Perhaps it is less often abolished or diminished, though in the maniacal bursts of ecstasy, where the sufferers present exalted pietism, intense love, burning adoration; where they shout forth hymns and petitions, or prostrate themselves in contemplation, absolved from all earthly ties, external impressions even of the most severe description are frequently unheeded. So, too, with muscular action. As a general rule, there is not that enormous development of muscular strength, that craving for violent exertion, and that endurance of fatigue, which have been so long recognised as characteristic of acute mania; but still there are many instances of mania ephemera which partake also of these characteristics, in which the disease seems to have generated new strength, in which dancing, leaping, and all sorts of extraordinary bodily contortions, are carried on, for hours together, without apparent inconvenience. How far such motions are automatic, and how far voluntary, it might be difficult to decide. The cerebral influences, however, determining motion in this disorder may acquire a force productive of spasm and convulsions. Garrick, liable to those temporary aberrations for which so many distinguished actors have been remarkable, after he had acted Lear or Othello, passed some hours in convulsions in bed. Where the manifestations already described are not present, there is most generally a muscular tremor, a tremulousness and uncertainty of muscular action, not unlike that observed in delirium tremens.

N. E., a young man of nervous temperament, whose case I have had recently under observation, was complaining of debility, occasional faintness, palpitation on slight exertion, coldness of the extremities, and loss of appetite, when one evening, after a fit of anger, produced by opposition to his wishes, he became suddenly insane. His relations were surprised at hearing strange and unaccountable noises proceeding from his room. On going to his apartment, they found the furniture in great disorder, and the young man himself in bed, laughing and chattering in the most unnatural manner. He at once recognised those who entered his room and named them, burst into tears, assured them that he was the victim of persecution, that there were men beneath his bed, and pointed out angels at the windows and in the room. He shouted, laughed, and sung, talked to imaginary beings, and insisted upon getting up and walking to a neighbouring churchyard. When prevented from doing so, he was irritated at first, but soon gave up the project. His hands shook, and his whole body shivered as if from cold. He complained of headache, and at his own desire had water poured over the head. This, he said, gave him relief; he ordered the light to be extinguished, as it hurt his eyes. After he had been ill about two hours, a dose of the sedative solution of opium was given him. In about three hours more he became calm and fell asleep. The next day he complained of lassitude and weariness, but mentally he was quite well.

The terrible alteration of countenance in ordinary mania which gives to the sufferer such a haggard and unnatural expression, even before the disease has begun to tell upon his strength, is not usually present in mania ephemera. Neither is the huskiness of voice, bristling of the hair, contraction of the skin of the forehead, or protruberance of the eyes, observable. The stools are not black and offensive, but the urine is generally loaded with phosphates, from the oxidation of the phosphorized fat of the nervous matter.

The symptoms of mania ephemera are invariably modified by the period of life and circumstances of the sufferer. When appearing in females, produced by influences operating through the generative focus of the cœnæsthesis, they usually partake of an hysterical character. The rapid evolution of the sexual organs and functions, or the derangement of these, sometimes induces morbid mental activity. Illustrative of this is the following case, with remarks, translated from Marc:—

"A female, subject at each catamenial period to mental disturbance, encountered, while thus affected, one of her own sex, whom she grossly insulted, in the presence of another person. The aggrieved party complained; the aggressor denied the fact, and the judge accepted her protestations of innocence upon oath, which was made in good faith, as she could recal nothing which occurred during these paroxysms of excitement. The complainant was found liable in expenses; but, discovering the witness of the injuries to which she had been exposed, and her declaration having been admitted, the falsehood of the original oath became evident.

"In consequence of this, Professor Berends, Frankfort sur l'Oder, was called upon to answer the following interrogatory: 'Is the state of the accused such as to call upon us to admit that her paroxysms of anger are such that she cannot recal what takes place during their continuance?'

"The report of the Professor was to the effect, 'that he had attentively studied the documentary evidence, and that he had personally, and in the presence of another medical man, investigated the sanitary condition of the accused. Surgeon L., who had professionally attended the woman, assured him that on the arrival of the menstrual period. and during the discharge, she was constantly attacked with an orgasm and cerebral congestion, with febrile acceleration of pulse, and that the exacerbation was ordinarily so violent as to be attended with delirium. During the epoch she became very irascible and subject to paroxysms of furious anger: her own statements corresponded closely with those of her medical attendant. Her external aspect and her general constitution clearly indicated an anormal impressibility, a great weakness of the nervous system, and an excessive irritability. The pupils were dilated, and her gaze, as well as her expression of countenance, could not leave any doubt as to her condition. Dr. B. did not hesitate to reply in the affirmative to the question proposed to him. It is certain that during a paroxysm of febrile delirium there is no recollection, or, at all events, an imperfect recollection of what has occurred, because the special senses are enfeebled or even perverted. Anger, as every other passion in excess, induces, it is known, an analogous condition, when, by the predominance of powerful and vivid impressions, the exercise of reflection aud volition are suspended. But in the case under consideration it should be remarked, that the disposition to anger, and to its most outrageous ebullitions, has its seat in the irritability and unhealthy tendencies of the nervous system, that consequently the origin of the fury should be attributed to the state of the body, inasmuch as when the excitement has reached a certain amount, it may be impossible for the accused to resist such an influence upon her moral nature. Besides, all medical men are of one opinion as to the accidents, such as spasms, convulsions, syncope, epilepsy, abdominal pain, with which menstruation may be complicated, and as to the disturbance in the cerebral functions which it may produce.

" 'This evacuation, it is true, is as natural to the female as pregnancy and parturition, during which temporary mental alienation often takes place. But, upon all occasions, when marked disorders attack the sex, we may rest assured that these are the consequence of an anormal or pathological condition. If, then, it be established that the accused abused the woman N. during the menstrual period, or near to it, and was suffering from the constitutional disturbance to which she is subject, it follows that she retained no knowledge of the insults complained of.' "*

The following case has been communicated to me by an eminent medical psychologist :—

"About ten years ago I attended a lady, who was described as recovering from an attack of mental excitement, of some duration, and which had been brought on by anxiety. She was labouring, when I saw her, under peculiarity and perversity of disposition. She recovered perfectly, and subsequently mingled freely and frequently in society, manifesting great gentleness of disposition, prudence, and self-possession. I was summoned to see her about two months since, and as the telegram indicated danger and the necessity for dispatch, I travelled as rapidly as railways and posters enabled me. The history of the case briefly was, that she had received an offer of marriage, under very perplexing circumstances, upon Thursday, which agitated her much, and rendered her sleepless ; that on Friday the catamenia appeared, and to such an alarming extent as apparently to amount to menorrhagia. After another sleepless night, she became, upon

---

* Marc, *De la Folie*, tom. ii. p. 512.

Saturday, hysterical, restless, loquacious, incoherent, and then maniacal. Though of fragile, delicate frame, her violence had been extreme, and had defied and exhausted the strength of five or six female servants. On the evening of Sunday the medical man in attendance exhibited two drachms of laudanum. In an hour or two she fell asleep, and when I arrived was still sleeping, but was restless, moaning, muttering, and had the aspect of complete exhaustion. Her sleep was attended with stertor, and had created alarm both in the medical man and the relatives. After sleeping for about ten hours, she awoke. She was still incoherent, disposed to talk, to toss her limbs about, and was evidently unable to recognise those about her, or where she was. There was, however, a great change in other respects; the pulse had fallen in frequency, and greatly in strength; the pupils were dilated: the skin was profusely covered with perspiration; the voice sunk to a whisper: the countenance was pale and collapsed; and various indications of increasing prostration were observed. Champagne was given immediately and freely; followed by jellies, soups, and other means of support and stimulation. The effect was seen at once; and in the course of a few hours tranquillity and repose and comparative intelligence were re-established; in a few days the patient was regarded by her friends as well, and after a short interval she resumed her former pursuits and her former place in society, without a trace of any mental or nervous affection, and without a suspicion on the part of those with whom she associated of the terrible ordeal through which she had passed."

Mania ephemera may occur during pregnancy, grafted upon the usual excitability of that state, and associated with its uncontrollable longings. It may also spring out of the cerebral congestions of the puerperal state and the period of lactation.

Many of the epidemic psychopathies which have from time to time appeared, seem to have been epidemics of ephemeral mania. They have almost without exception had their source in emotions, in an intense regard for religion, morality, the fine arts, or in an uncouth fear of the supernatural and unseen. They have been disseminated by psychical contagion and pathological sympathy, and have taken root and grown to rankness in the soil of ignorance, superstition, and nervous debility. They have also withered and vanished before moral forces. When the intellectual feeling, which in its normal exercise is termed enthusiasm, oversteps the boundaries of reason, it gives origin to innumerable extravagant acts and ideas, which can only be regarded as constituting a brief paroxysm of mania. The madness of the Milesian maidens, described by Plutarch, cured by an edict enforcing public exposure upon subsequent sufferers; the dancing epidemics of the Middle Ages, recounted by Hecker, the prevalent madness of the nuns of Saxony and Brandenburg, recorded by Simon Goulard, in which these ladies "predicted, capered, climbed up walls, spoke various languages, bleated like sheep, and amused themselves by biting each other;"—the insanity of the Amsterdam foundlings, the *ex-stasis religiosa* of the Swedes; the ecstasies of primitive races —all afford examples of ephemeral mania occurring epidemically. The phenomena of these conditions, which deserve a full and discriminating consideration such as cannot here be given them, are in fact but the symptoms of mania, modified by prevailing notions and beliefs. Those afflicted by them, like maniacs, had all their vital endowments deranged, and had lost the capacity of reasoning, of comparing, and associating their ideas. Memory had deserted them, volition was in abeyance, and all the functions of organic life were more or less disturbed.

It is not my purpose to enlarge further upon this topic, or upon the treatment of mania ephemera, which has, however, only to be conducted upon the general principles of medical science. It can be scarcely necessary to observe that during the paroxysm the patient must be confined in a large, well-ventilated, partially darkened room, or only allowed to take exercise under the most watchful supervision; that constraint must be avoided, but the most rigorous, though mild superintendence observed. Equanimity, with calm decision, must characterize all intercourse with the patient, whose relations should be kept away from him. Great benefit will generally accrue from the use of evaporating lotions applied to the head, or from cold affusion on the head while the body is in a warm bath. Mild purgatives should be administered, and ten to twenty minims of the sedative solution of opium with tincture of hyoscyamus, or a quarter of a grain to a grain of muriate of morphia in camphor mixture. These are invaluable in allaying restlessness and irritability, and in inducing sleep, so often the conclusion of an attack of ephemeral mania. The efficacy of digitalis is not yet established. Wine is sometimes required, and when the heart's action is feeble, must be fearlessly given, even when violent excitement exists.

Both during and after the attack the state of the system must receive the most careful attention. Existing diseases must be treated, and the constitution must be strengthened by every possible means. The surest prophylaxis against a return of the ailment is founded upon a consideration of its predisposing and exciting causes; and thus it is that in this ailment restraint of caprices, passions, and selfish feelings should be so forcibly inculcated. Among the medical agents valuable in preventing relapse, quinine occupies the foremost place; its power being probably dependent upon its properties of restoring deficient tone to the vascular system and of removing congestions wherever these exist. Next to quinine, the preparations of iron appear to be of most use. But, indeed, there are no peculiarities in the treatment of mania ephemera. Regard being had to the causes of the disease, to the degree of excitement and vascular fulness, and to the state of the secretions, excretions, and reproductive functions, each case has only to be conducted upon ordinary hygienic and pharmaceutical principles.

---

### Art. V.—MORELL'S INDUCTIVE MENTAL PHILOSOPHY.*

